# Brassinin inhibits STAT3 signaling pathway through modulation of PIAS-3 and SOCS-3 expression and sensitizes human lung cancer xenograft in nude mice to paclitaxel

**DOI:** 10.18632/oncotarget.3443

**Published:** 2015-01-31

**Authors:** Jong Hyun Lee, Chulwon Kim, Gautam Sethi, Kwang Seok Ahn

**Affiliations:** ^1^ College of Korean Medicine, Kyung Hee University, Hoegidong Dongdaemungu, Seoul, Republic of Korea; ^2^ Department of Pharmacology, Yong Loo Lin School of Medicine, National University of Singapore, Singapore

**Keywords:** Brassinin, STAT3, PIAS-3, SOCS-3, apoptosis

## Abstract

Persistent phosphorylation of signal transducers and activators of transcription 3 (STAT3) is frequently observed in tumor cells. We found that brassinin (BSN) suppressed both constitutive and IL-6-inducible STAT3 activation in lung cancer cells. Moreover, BSN induced PIAS-3 protein and mRNA, whereas the expression of SOCS-3 was reduced. Knockdown of PIAS-3 by small interfering RNA prevented inhibition of STAT3 and cytotoxicity by BSN. Overexpression of SOCS-3 in BSN-treated cells increased STAT3 phosphorylation and cell viability. BSN down-regulated STAT3-regulated gene products, inhibited proliferation, invasion, as well as induced apoptosis. Most importantly, when administered intraperitoneally, combination of BSN and paclitaxel significantly decreased the tumor development in a xenograft lung cancer mouse model associated with down-modulation of phospho-STAT3, Ki-67 and CD31. We suggest that BSN inhibits STAT3 signaling through modulation of PIAS-3 and SOCS-3, thereby attenuating tumor growth and increasing sensitivity to paclitaxel.

## INTRODUCTION

Signal transducer and activator of transcription 3 (STAT3) belongs to the STAT family of proteins, which is an inducible transcription factor in the cytoplasm of most cell types. STAT3 can integrate signals from various extracellular stimuli and kinase pathways, and it is hence regulating many critical functions in human normal and malignant tissues, such as differentiation, proliferation, survival, angiogenesis, and immune system regulation [1, 2]. Activation of STAT3 is also known to convey a variety of survival signals by up-regulating the expression of genes involved in cell cycle progression (*Cyclin D1*), angiogenesis (VEGF, HIF-1 α), cell migration (MMP-2/9), immune evasion (RANTES), and anti-apoptotic genes (*Bcl-2, Bcl-xL, Survivin*) [3-5].

Constitutive activation of STAT3 has been observed in 22%-65% of non-small cell lung cancers (NSCLC) [6, 7]. Jiang *et al* showed that positive phospho-STAT3 expression was detected in 82 of the 127 carcinomas (64.6%) but in only 21 of the 56 normal tissue samples (37.5%) and phospho-STAT3 immunoreactivity was significantly correlated with sex (*p =* 0.004), smoking history (*p =* 0.006), EGFR mutation status (*p =* 0.003), clinical stage (*p =* 0.034), and lymph node metastasis (*p =* 0.009) [8]. Xu *et al* used a meta-analysis to quantitatively assess STAT3 and phospho-STAT3 expression on the prognosis of NSCLC and found that high STAT3 or phospho-STAT3 expression is a strong predictor of poor prognosis among patients with NSCLC [9]. Collectively, these data suggest that aberrant STAT3 activation is a strong predictor of poor prognosis in patients with NSCLC.

There are two group of signaling proteins known to inactivate STAT proteins, the protein inhibitors of activated STAT (PIAS) [10] and the suppressors of cytokine signaling (SOCS) [11-13]. Two proteins are known to participate in the negative regulation of the STAT signaling pathway [14]. Interestingly, PIAS-3 belongs to a multi-gene family which was first identified as a transcriptional repressor of activated STAT3 that blocks transactivation of a STAT3-responsive reporter gene and inhibition of the STAT3 DNA-binding activity [10]. High PIAS-3 expression has been observed in various human cancer, such as lung, breast, and brain tumors [15]. PIAS-3 overexpression can suppress cell growth in human lung tumor cells [16] and is associated with apoptosis in prostate cancer cells [17]. SOCS-3 inhibits phosphorylation of STAT3 via binding to JAK-proximal sites on cytokine receptors to suppress JAK activity [18]. Additionally, SOCS-3 is not only an intracellular blocker of STAT3 but also a STAT3 transcriptional target [19].

In this study, we analyzed the potential chemosenstizing effect(s) of brassinin (BSN), a phytoalexin first identified as a constituent of cabbage, that has been reported to possess chemopreventive [20], antiproliferative [21, 22], antifungal [23], and anticarcinogenic [24, 25] activities against human lung carcinoma. This agent has exhibited cancer chemopreventive activity in mouse models of mammary and skin carcinogenesis [26], exerted remarkable anti-proliferative effects on the human cervical HeLa, human epithelial A431, and human breast MCF7 cancer cells [27], and exerted pro-apoptotic effects against human colorectal cancer cells [25]. Also, BSN is known to act as a potent chemopreventive agent through the induction of phase II drug-metabolizing enzymes [28]. More specifically, BSN has been reported to induce G1 phase arrest through increase of p21 and p27 by inhibition of the phosphatidylinositol 3-kinase signaling pathway [25] and our laboratory has demonstrated that BSN can also suppress the constitutive activation of PI3K/Akt/mTOR/S6K1 signaling cascade [29]. Although various oncogenic targets as discussed above have been described to account for the potent anticancer activities of BSN, our study is the first one to explore the effects of BSN both on STAT3 signaling pathway and on the negative regulators of STAT3 signaling (PIAS-3 and SOCS-3) in human lung carcinoma. We found that BSN suppressed both constitutive and IL-6-inducible STAT3 activation; down-regulated STAT3-regulated gene products; and potentiated paclitaxel-induced apoptotic effects in NSCLC both *in vitro* and *in vivo*.

## RESULTS

**FIGURE 1:**
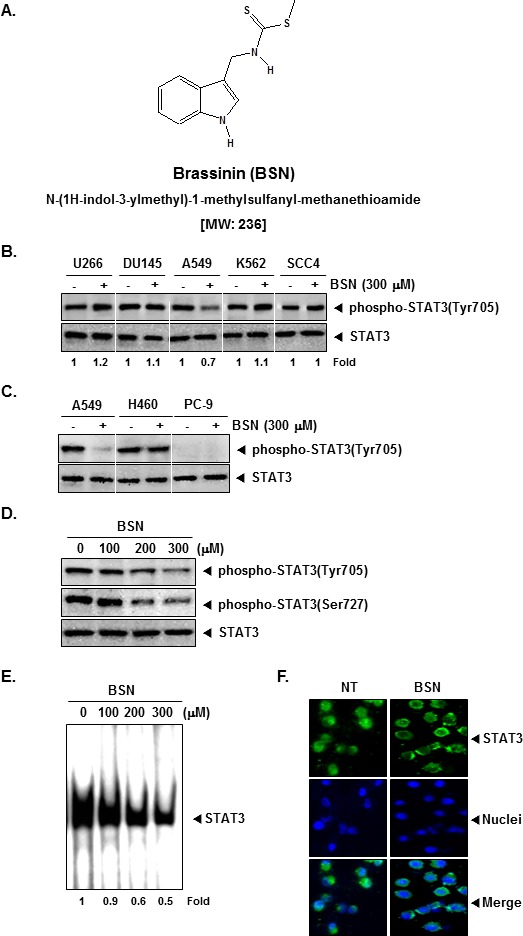
BSN inhibits constitutively active STAT3 in A549 cells (A) The chemical structure of brassinin (BSN). (B) U266, DU145, A549, K562, and SCC4 cells (1 × 10^6^ cells/well) were treated with 300 μM of BSN for 4 h. Whole-cell extracts were prepared and immunoblotted with antibodies for phospho-STAT3 (Tyr705) and STAT3. (C) A549, H460, and PC-9 cells (1 × 10^6^ cells/well) were treated with 300 μM of BSN for 4 h. Whole-cell extracts were prepared and immunoblotted with antibodies for phospho-STAT3 (Tyr705) and STAT3. (D) A549 cells (1 × 10^6^ cells/well) were treated with the indicated concentrations of BSN for 4 h. Whole-cell extracts were prepared and immunoblotted with antibodies for phospho-STAT3 (Tyr705), phospho-STAT3 (Ser727), and STAT3. (E) A549 cells (1 × 10^6^ cells/well) were treated with the indicated concentrations of BSN for 4 h and analyzed for nuclear STAT3 levels by EMSA. (F) BSN causes inhibition of translocation of STAT3 to the nucleus. A549 cells (4 × 10^4^ cells/well) were incubated with or without 300 μM BSN for 4h and then analyzed for the intracellular distribution of STAT3 by immunocytochemistry. The results shown here are representative of three independent experiments.

The goals of this study were, first, to determine whether BSN exerts the anti-cancer effects through the abrogation of the STAT3 signaling pathway in NSCLC cells; second, to analyze whether BSN can enhance the antitumor effects of paclitaxel, chemotherapeutic drug used extensively to treat NSCLC patients; third, to investigate whether BSN potentiates the effects of these targeted therapies *in vivo*. The chemical structure of BSN is shown in (Fig. 1A).

### BSN specifically inhibits constitutive STAT3 activation in A549 cells, but not in U266, DU145, K562, and SCC4 cells

We first investigated whether BSN can modulate constitutive STAT3 activation in a variety of human cancer cell lines. Because U266, DU145, A549, K562, and SCC4 cells have been shown to express constitutive STAT3 activation, we set out to determine whether BSN could inhibit this activation in these cells. Interestingly we found that it did inhibit STAT3 activation only in A549 cells, but not in U266, DU145, K562, and SCC4 cells (Fig. 1B, upper panel) and had no effect on the expression of STAT3 proteins (Fig. 1B, lower panel), thereby indicating that BSN-induced suppression of STAT3 phosphorylation is cell type-specific.

### BSN specifically inhibits constitutive STAT3 activation in A549 cells, but not in several human lung cancer cell lines

We next investigated the ability of BSN can modulate constitutive STAT3 activation in a variety of human lung cancer cell lines. As shown in (Fig. 1C), interestingly, A549 and H460 cells express high levels of phospho-STAT3 protein, but PC-9 cells did not show detectable phospho-STAT3. We also found that the constitutive activation of STAT3 was suppressed by BSN in A549 cells, but not in H460 cells. The data suggest that inhibition of STAT3 activation by BSN of cell-type specific and BSN had little effect on the expression of total STAT3 proteins.

### BSN suppresses constitutive STAT3 phosphorylation in a concentration-dependent manner

The ability of BSN to modulate constitutive STAT3 activation in A549 cells in a dose-dependent manner was investigated. BSN suppressed the phosphorylation of STAT3 at both (Tyr705 and Ser727 residues) in a concentration-dependent manner in A549 cells. BSN had no effect on the expression of STAT3 proteins (Fig. 1D).

### BSN inhibits binding of STAT3 to the DNA

Because tyrosine phosphorylation causes the dimerization of STAT3 and their translocation to the nucleus, where they bind to DNA and regulate gene transcription, we determined whether BSN suppresses the DNA binding activity of STAT3. EMSA analysis of nuclear extracts prepared from A549 cells showed that BSN substantially inhibited STAT3-DNA binding activity in a concentration-dependent manner (Fig. 1E). These results show that BSN can abrogate the DNA binding ability of STAT3.

### BSN reduces nuclear pool of STAT3 in NSCLC cells

Because the active dimer of STAT3 is capable of translocating to the nucleus and inducing transcription of specific target genes, we analyzed whether BSN suppresses the nuclear translocation of STAT3. Immunocytochemistry (Fig. 1F) clearly demonstrate that BSN blocked the translocation of STAT3 into the nucleus in A549 cells.

### BSN suppresses constitutive activation of JAK1, JAK2, and Src

**FIGURE 2:**
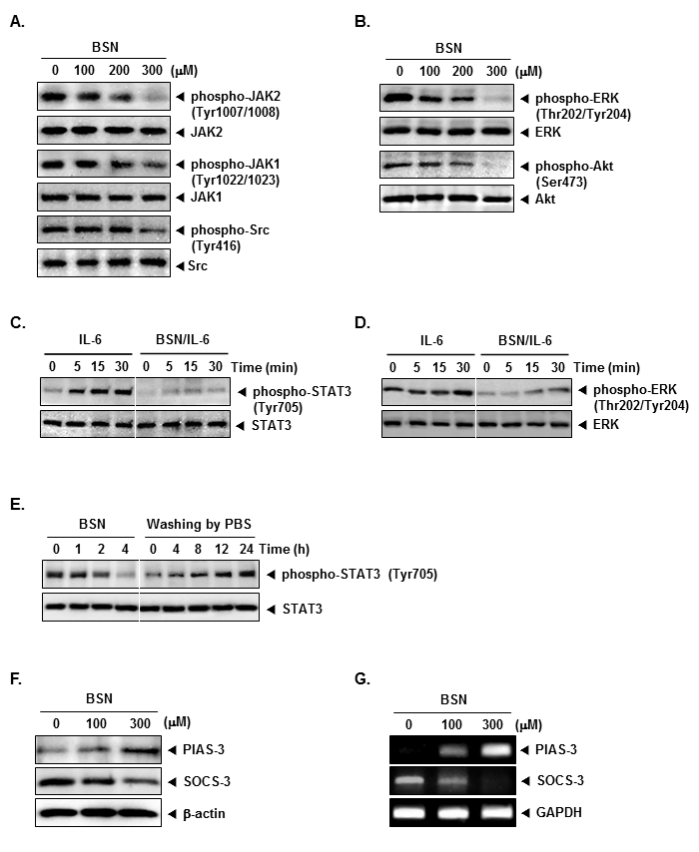
BSN suppresses the activation of JAK1/2 and Src in a dose-dependent manner (A) A549 cells (1 × 10^6^ cells/well) were treated with indicated concentrations of BSN, after which whole-cell extracts were prepared and 20 μg portions of those extracts were resolved on 8% SDS-PAGE gel, electrotransferred onto nitrocellulose membranes, and probed with phospho-JAK1 (Tyr1022/1023), phospho-JAK2 (Tyr1007/1008), and phospho-Src (Tyr416) antibodies. The same blots were stripped and reprobed with JAK1, JAK2, and Src antibody to verify equal protein loading. (B) A549 cells (1 × 10^6^ cells/well) were treated with indicated concentrations of BSN, after which whole-cell extracts were prepared and 20 μg portions of those extracts were resolved on 8% SDS-PAGE gel, electrotransferred onto nitrocellulose membranes, and probed with phospho-ERK (Thr202/Tyr204) and phospho-Akt (Ser473) antibodies. The same blots were stripped and reprobed with ERK and Akt antibody to verify equal protein loading. (C) H1299 cells (1 × 10^6^ cells/well) were treated with 300 μM of BSN for 4 h and then stimulated with IL-6 (25 ng/ml) for the indicated time. Whole-cell extracts were prepared and immunoblotted with antibodies for phospho-STAT3 (Tyr705) and STAT3. (D) H1299 cells (1 × 10^6^ cells/well) were treated with 300 μM of BSN for 4 h and then stimulated with IL-6 (25 ng/ml) for the indicated time. Whole-cell extracts were prepared and immunoblotted with antibodies for phospho-ERK (Thr202/Tyr204) and ERK. (E) A549 cells (1 × 10^6^ cells/well) were treated with 300 μM of BSN for the indicated durations or treated for 4 h and washed with PBS twice to remove BSN before resuspension in fresh medium. Cells were removed at indicated times and lysed to prepare the whole-cell extract. Twenty micrograms of whole-cell extracts were resolved on 8% SDS-PAGE, electrotransferred onto nitrocellulose membrane, and probed with phospho-STAT3 (Tyr705) and STAT3 antibodies. (F) A549 cells (1 × 10^6^ cells/well) were treated with the indicated concentrations of BSN for 4 h. Whole-cell extracts were prepared and immunoblotted with antibodies for SOCS-1 and SOCS-3. The same blots were stripped and reprobed with β-actin antibody to verify equal protein loading. (G) A549 cells (1 × 10^6^ cells/well) were treated with the indicated concentrations of BSN for 4 h. Total RNA was extracted and examined for expression of SOCS-1 and SOCS-3 by Reverse Transcription-Polymerase Chain Reaction (RT-PCR). Glyceraldehyde-3-phosphate dehydrogenase (GAPDH) was used as an internal control to show equal RNA loading. The results shown here are representative of three independent experiments.

STAT3 has been reported to be activated by the soluble tyrosine kinases of the Janus family (JAK). Because JAK1 and JAK2 were the main upstream kinases involved, we examined the effect of BSN on JAK1 and JAK2 activation. As shown in (Fig. 2A), both JAK1 and JAK2 were constitutively active in A549 cells and the treatment with BSN clearly suppressed this phosphorylation in a concentration-dependent manner. In addition, STAT3 is also activated by soluble tyrosine kinases of the Src kinase families. We determined the effect of BSN on the constitutive activation of Src kinase in A549 cells. We found that BSN also suppressed the constitutive phosphorylation of c-Src kinase (Fig. 2A).

### BSN inhibit constitutive activation of ERK and Akt

We next investigated whether BSN affects constitutive activation of ERK (Thr202/Tyr204) in A549 cells. We found that BSN suppressed the constitutive phosphorylation of ERK (Thr202/Tyr204), but had no effect on the expression of ERK proteins (Fig. 2B). Activation of Akt has also been linked with STAT3 activation. We therefore investigated whether BSN modulates constitutive activation of Akt (Ser473) in A549 cells. We found that BSN attenuated the constitutive phosphorylation of Akt (Ser473). BSN had no effect on the expression of Akt proteins (Fig. 2B).

### BSN also inhibits IL-6-induced STAT3 and ERK phosphorylation

Because IL-6 is a growth factor for NSCLC cells and induces STAT3 and ERK phosphorylation, we determined whether BSN could inhibit IL-6-induced STAT3 and ERK phosphorylation. H1299 cells, which lack constitutively active STAT3 and ERK, were treated with IL-6 for different times and then examined for phosphorylated STAT3 and ERK. IL-6-induced phosphorylation of both STAT3 and ERK proteins in a time-dependent manner in H1299 cells. However, in cells pretreated with BSN for 4 h, IL-6-induced STAT3 and ERK phosphorylation was suppressed clearly (Fig. 2C and D).

### BSN-induced inhibition of STAT3 phosphorylation is reversible

We further examined whether BSN-induced inhibition of STAT3 phosphorylation is reversible. A549 cells were first treated for various intervals with BSN and then washed twice with PBS to remove the agent. The cells were then cultured in fresh medium for various durations, and the level of phosphorylated STAT3 was observed. BSN-induced the suppression of STAT3 phosphorylation (Fig. 2E, left), but after the removal of BSN, phosphorylated STAT3 gradually increased (Fig. 2E, right). The reversal was complete by 24 h and did not involve any changes in STAT3 protein levels (Fig. 2E, bottom).

### BSN induces the expression of PIAS-3 and attenuates the expression of SOCS-3 in A549 cells

The SOCS (suppressors of cytokine signaling) proteins and PIAS (protein inhibitors of activated STAT) have been suggested to function as inhibitors of cytokine receptor signaling. We examined whether BSN can modulate the expression of SOCS-3 and PIAS-3 in A549 cells. We found BSN led to an increased expression of PIAS-3 and decreased expression SOCS-3 at the protein level (Fig. 2F). Also, we found that treatment of BSN enhanced the expression of PIAS-3 and attenuated the expression of SOCS-3 at the mRNA level (Fig. 2G).

### Silencing of PIAS-3 or overexpression of SOCS-3 in BSN-treated cells reverse the effect of BSN on activated STAT3 and cell viability

**FIGURE 3:**
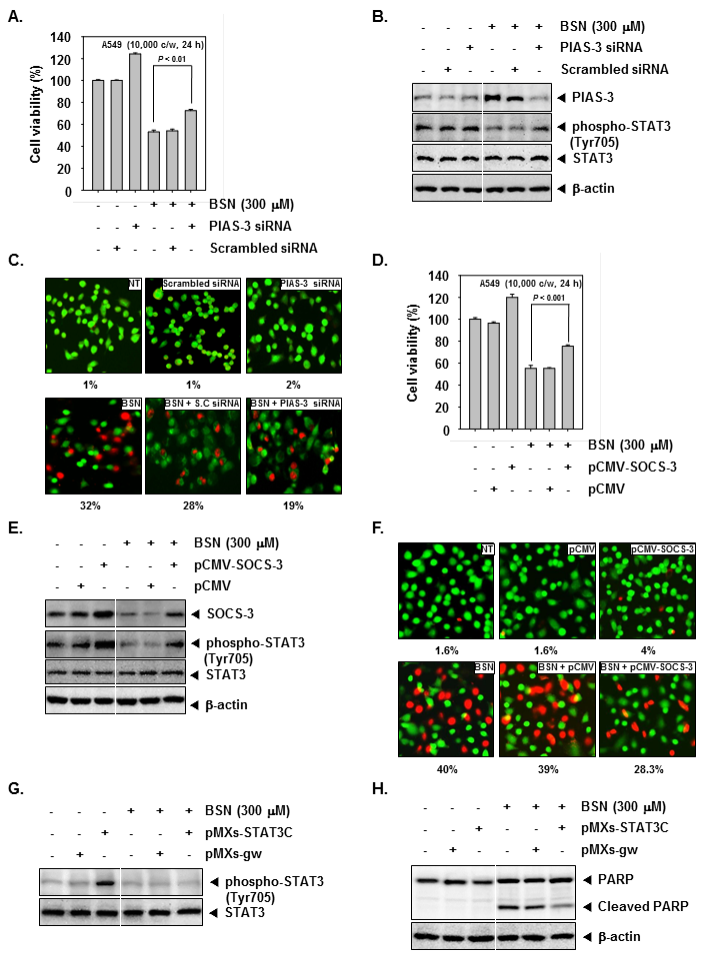
Silencing of PIAS-3 and ectopic expression of SOCS-3 in BSN-treated cells reverse the effect of BSN on STAT3 activation and cell viability A549 cells were transiently transfected with siRNA against PIAS-3. (A) Transiently transfected cells were treated with BSN (300 μM) for 24 h. Cell viability was measured by MTT assay. Values represent the mean ± SD of triplicate cultures (**P <0.01). (B) Transiently transfected cells were treated with 300 μM of BSN for 4 h. Then, equal amounts of lysate were analyzed by Western blot analysis using antibodies against PIAS-3, phospho-STAT3 (Tyr705), and STAT3. The same blots were stripped and reprobed with β-actin antibody to verify equal protein loading. (C) Transiently transfected cells were treated with BSN (300 μM) for 24 h. Cells were stained with a live/dead assay reagent for 30 minutes and then analyzed under a fluorescence microscope as described in “Materials and methods.” Percentage of apoptosis is indicated in the inset. A549 cells were transiently transfected with pCMV-SOCS-3 or pCMV (control vector) plasmid. SOCS-3 protein was overexpressed in pCMV-SOCS-3 tranfected A549 cells compared to control. (D) Transiently transfected cells were treated with BSN (300 μM) for 24 h. Cell viability was measured by MTT assay. Values represent the mean ± SD of triplicate cultures (***P <0.001). (E) Transiently transfected cells were treated with 300 μM of BSN for 4 h. Then, equal amounts of lysate were analyzed by Western blot analysis using antibodies against SOCS-3, phospho-STAT3 (Tyr705), and STAT3. The same blots were stripped and reprobed with β-actin antibody to verify equal protein loading. (F) Transiently transfected cells were treated with BSN (300 μM) for 24 h. Cells were stained with a live/dead assay reagent for 30 minutes and then analyzed under a fluorescence microscope as described in “Materials and methods.” Percentage of apoptosis is indicated in the inset. (G) MEF cells were transiently transfected with pMXs-STAT3C or pMXs-gw (control vector) plasmid. STAT3C protein was overexpressed in pMXs-STAT3C tranfected MEF cells compared to control. Transiently transfected cells were treated with 300 μM of BSN for 4 h. Then, equal amounts of lysate were analyzed by Western blot analysis using antibodies against phospho-STAT3 (Tyr705) and STAT3. (H) Equal amounts of lysate were analyzed by Western blot analysis using antibody against PARP. The same blots were stripped and reprobed with β-actin antibody to verify equal protein loading. The results shown here are representative of three independent experiments.

Our results demonstrate that increased expression of PIAS-3 and decreased SOCS-3 expression following BSN treatment is associated with reduced phospho-STAT3 (Tyr705) expression and decreased cell viability. To explore if silencing PIAS-3 and over-expressing of SOCS-3 would reverse the effect of BSN on STAT3 activation, A549 cells were treated with or without BSN (300 μM) and transfected with siRNA against PIAS-3 or with pCMV-SOCS-3 plasmid to inhibit PIAS-3 and up-regulate SOCS-3 expression, respectively. Transfection of BSN-treated cells with siRNA of PIAS-3 abrogated BSN-induced cell growth inhibition, whereas a significant increase in cell viability was observed when non-treated (NT) cells were transfected with siRNA of PIAS-3 (Fig. 3A). In A549 cells, siRNA against PIAS-3 inhibited PIAS-3 expression in BSN-treated and non-treated transfected cells compared to scrambled siRNA-transfected cells. A marked decrease of phospho-STAT3 (Tyr705) was observed in BSN-treated cells. Interestingly, a marked increase of phospho-STAT3 (Tyr705) was seen in BSN-treated A549 cells transfected with siRNA of PIAS-3 (Fig. 3B). The Live and Dead assay (which measures intracellular esterase activity and plasma membrane integrity) showed that BSN-treated cells transfected with PIAS-3 siRNA showed a decrease in apoptosis from 32 to 19% (Fig. 3C). Transfection of BSN-treated cells with pCMV-SOCS-3 plasmid abrogated BSN-induced cell growth inhibition, whereas a significant increase in cell viability was observed when non-treated (NT) cells were transfected with pCMV-SOCS-3 plasmid (Fig. 3D). Transfection of pCMV-SOCS-3 plasmid resulted in the upregulation of SOCS-3 expression in BSN-treated and non-treated transfected cells compared to control plasmid-transfected cells. A marked decrease of phospho-STAT3 (Tyr705) was observed in BSN-treated cells. Interestingly, a marked increase of phospho-STAT3 (Tyr705) was seen in BSN-treated A549 cells transfected with pCMV-SOCS-3 plasmid (Fig. 3E). The Live and Dead assay showed that BSN-treated cells upon transfection with SOCS-3 plasmid siRNA resulted in reduction of apoptosis from 40 to 28.3% (Fig. 3F).

### Overexpression of STAT3 attenuates BSN-mediated apoptosis

We investigated whether overexpression of STAT3 by pMXs-STAT3C plasmid can prevent the effects of BSN. The cells transfected with by pMXs-STAT3C clearly showed overexpression of phospho-STAT3 (Tyr705) as compared with those transfected with only control plasmid, and the overexpression of STAT3 was clearly inhibited by BSN treatment in MEF cells (Fig. 3G). As shown in (Fig. 3H), overexpression of STAT3 led to the attenuation of BSN-mediated cleavage of PARP as compared to the control, indicating that STAT3 is one of the major molecular targets involved in BSN-induced apoptosis.

### BSN suppresses cell proliferation in human lung cancer cells

**FIGURE 4:**
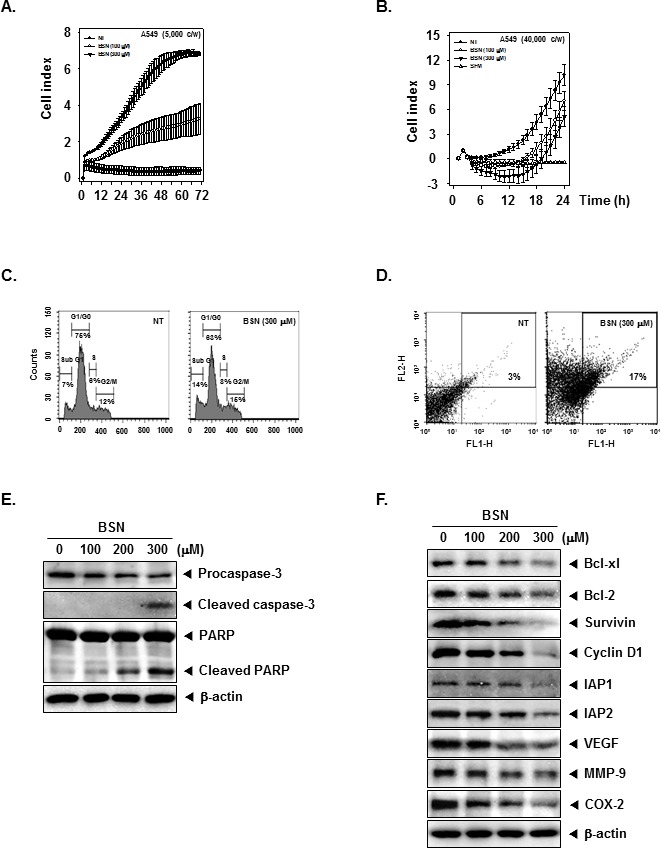
BSN suppresses cell proliferation, invasion, and induced apoptosis in A549 cells (A) Cell proliferation assay was performed using the Roche xCELLigence Real-Time Cell Analyzer (RTCA) DP instrument (Roche Diagnostics GmbH, Germany) as described under “Material and methods”. A549 cells (5 × 10^3^ cells/well) were seeded onto 16-well E-plates and continuously monitored using impedance technology. (B) Invasion assay was performed using the Roche xCELLigence Real-Time Cell Analyzer (RTCA) DP instrument (Roche Diagnostics GmbH, Germany) as described under ‘Materials and methods’. We tested the effect of BSN on A549 cell invasive activity (4 × 10^4^ cells/well) in the matrigel-coated CIM (cellular invasion/migration)-plate 16 exposed to various indicated concentrations of BSN. (C) A549 cells (1 × 10^6^ cells/well) were seeded onto 6-well plates, they were treated with 300 μM of BSN for 24 h. Then, the cells were harvested, washed with a cold PBS buffer, and digested with RNase A. Cellular DNA staining with propidium iodide and flow cytometric analysis was done to determine the cell cycle distribution as described in “Materials and methods”. (D) A549 cells (1 × 10^6^ cells/well) were treated with 300 μM of BSN for 24 h. The cells were incubated with an FITC-conjugated annexin V antibody and then analyzed by a flow cytometry as described in “Materials and methods”. (E) A549 cells (1 × 10^6^ cells/well) were treated with indicated concentrations of BSN, after which whole-cell extracts were prepared and 20 μg portions of those extracts were resolved on 10% SDS-PAGE gel, electrotransferred onto nitrocellulose membranes, and probed against caspase-3 and PARP antibodies. The same blots were stripped and reprobed with β-actin antibody to verify equal protein loading. (F) A549 cells (1 × 10^6^ cells/well) were treated with indicated concentrations of BSN, after which whole-cell extracts were prepared and 20 μg portions of those extracts were resolved on 10% SDS-PAGE gel, electrotransferred onto nitrocellulose membranes, and probed against Bcl-xl, Bcl-2, Survivin, Cyclin D1, IAP1, IAP2, VEGF, MMP-9, and COX-2 antibodies. The same blots were stripped and reprobed with β-actin antibody to verify equal protein loading. The results shown here are representative of three independent experiments.

To specifically examine the anti-tumor activity of BSN on A549 cells, the cells were treated with the indicated concentrations of BSN, and then cell viability was analyzed every 15 min time intervals using the xCELLigence RTCA MP Instrument (Roche Diagnostics GmbH, Germany). As shown in (Fig. 4A), BSN significantly suppressed cell proliferation in A549 cells in a dose and time-dependent manner.

### BSN suppresses lung cancer cell invasive activity

Whether BSN can modulate A549 lung cancer cell invasion activity was investigated. To determine this, A549 cells were seeded to the matrigel (BD Biosciences, Becton-Dickinson, Franklin Lakes, NJ)-coated CIM-Plate 16 with or without BSN and then examined for invasion. As shown in (Fig. 4B), BSN significantly suppressed tumor cell invasion activity in the cells.

### BSN causes the increased accumulation of the cells in the sub-G1 phase in A549 cells

We set out to determine the effect of BSN on cell cycle phase distribution. Importantly, we also found that the sub-G1 contents of DNA standing for apoptotic portions were significantly increased in BSN-treated A549 cells. BSN increased the cell accumulation in the sub-G1 phase (14%) compared with the non-treated (NT) cells (7%) (Fig. 4C). Taken together, these results suggest that BSN induced apoptotic cell death in A549 cells.

### BSN promotes apoptotic cell death in A549 cells

To evaluate the potential activities of BSN to induce apoptosis, we performed the annexin V assay. BSN increased early apoptotic cells in A549 cells in the annexin V assay and reached up to 17% at a concentration of 300 μM compared with the non-treated (NT) cells (3%) (Fig. 4D).

### BSN activates caspase-3 and causes PARP cleavage

Whether suppression of constitutively active STAT3 in A549 cells by BSN leads to apoptosis was also investigated. A549 cells were treated with various concentration of BSN and then were examined for Caspase-3 activation by Western blotting using specific antibody. We found a concentration-dependent activation of caspase-3 by BSN. Activation of downstream caspase-3 led to the cleavage of a 116 kDa PARP protein into 87 kDa fragments. These results clearly suggest that BSN induces Caspase-3-dependent apoptosis in A549 cells (Fig. 4E).

### BSN down-regulates the expression of various proteins involved in apoptosis

STAT3 activation has been shown to regulate the expression of various gene products involved in cell survival, proliferation, and angiogenesis. We observed that the expression of the cell cycle regulator protein Cyclin D1; anti-apoptotic proteins Bcl-xl, Bcl-2, IAP1, IAP2, and Survivin; angiogenic gene product VEGF; metastatic gene product MMP-9; and the inflammatory protein COX-2, all reported to be regulated by STAT3, was modulated upon BSN treatment. BSN treatment down-regulated the expression of these proteins in a concentration-dependent manner (Fig. 4F).

### BSN enhances the effect of paclitaxel on induction of apoptosis in NSCLC cells

**FIGURE 5:**
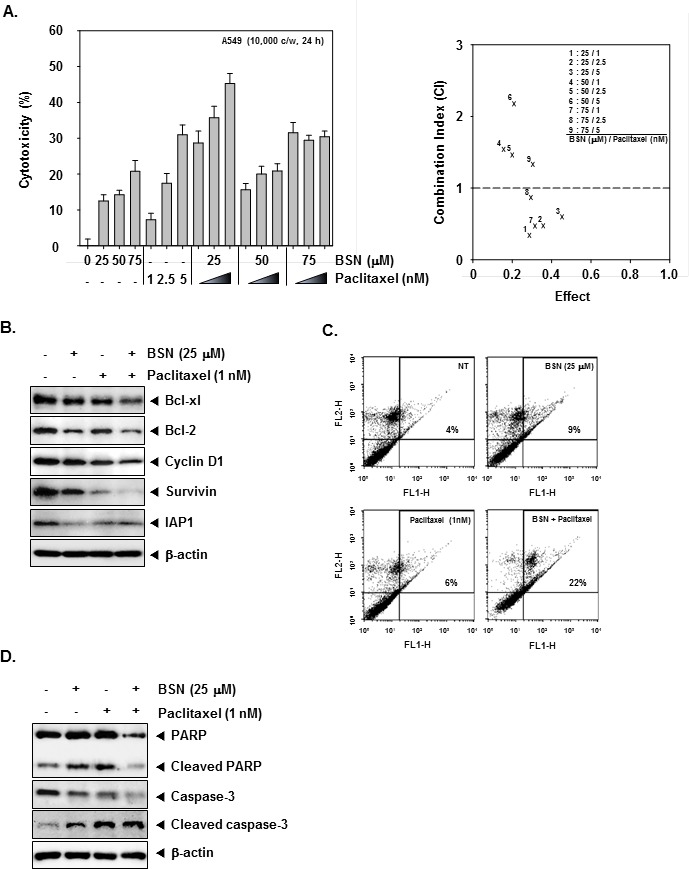
BSN potentiates the cytotoxic and apoptotic effects of targeted therapies in A549 cells (A) A549 cells (1× 10^4^ cells/well) were treated with BSN (0, 25, 50, and 75 μM), and paclitaxel (0, 1, 2.5, and 5 nM) for 24 h. The cytotoxicity was determined by MTT assays (*left*). BSN synergistically enhances paclitaxel-induced cell death in A549 cells (*right)*. The average of the CI values obtained at nine different combinations. CI of less than 1 was considered synergistic; CI of 1 was considered additive and a CI greater than 1 antagonistic. (B) A549 cells (1 × 10^6^ cells/well) were co-treated with 25 μM of BSN and 1 nM of paclitaxel for 24 h. After which whole-cell extracts were prepared and 20 μg portions of those extracts were resolved on 10% SDS-PAGE gel, electrotransferred onto nitrocellulose membranes, and probed against Bcl-xl, Bcl-2, Cyclin D1, Survivin, and IAP1 antibodies. The same blots were stripped and reprobed with β-actin antibody to verify equal protein loading. The results shown here are representative of three independent experiments. (C) A549 cells (1 × 10^6^ cells/well) were co-treated with 25 μM of BSN and 1 nM paclitaxel for 24 h. The cells were incubated with an FITC-conjugated annexin V antibody and then analyzed by a flow cytometry as described in “Materials and methods”. (D) A549 cells (1 × 10^6^ cells/well) were co-treated with 25 μM of BSN and 1 nM paclitaxel for 24 h. After which whole-cell extracts were prepared and 20 μg portions of those extracts were resolved on 10% SDS-PAGE gel, electrotransferred onto nitrocellulose membranes, and probed against caspase-3 and PARP antibodies. The same blots were stripped and reprobed with β-actin antibody to verify equal protein loading. The results shown here are representative of three independent experiments.

Currently, paclitaxel is a mitotic inhibitor that is being used in cancer chemotherapy. We set out to determine whether BSN can enhance paclitaxel-induced cell death. First, we determined whether a combination of BSN and paclitaxel can modulate the growth of A549 cells exposed to various concentrations of these compounds for 24 h. As shown in (Fig. 5A), the CIs indicated that certain combinations of BSN and paclitaxel (i.e. 25 μM BSN/1 nM paclitaxel) synergistically inhibited A549 cell growth. Next, we found that BSN or paclitaxel alone at sub-optimal concentrations had little effect on levels of Bcl-xl, Bcl-2, Cyclin D1, Survivin, and IAP1 proteins in A549 cells. However, treatment of cells with the combination of BSN and paclitaxel resulted in a marked attenuation in the expression levels of all of these proteins (Fig. 5B). Also, we further examined whether BSN can potentiate the apoptotic effect of paclitaxel in A549 cells by annexin V assay. As shown in (Fig. 5C), BSN substantially enhanced the apoptotic effects of paclitaxel in A549 cells. In addition Caspase-3 and PARP cleavage were further increased by the co-treatment of BSN along with paclitaxel rather than treatment with individual agents alone in A549 cells (Fig. 5D). Overall, these results show that combined treatment with BSN and paclitaxel increased apoptosis in A549 cells as compared with either drug alone.

### BSN potentiates the antitumor effects of paclitaxel in a xenograft lung cancer mouse model

**FIGURE 6:**
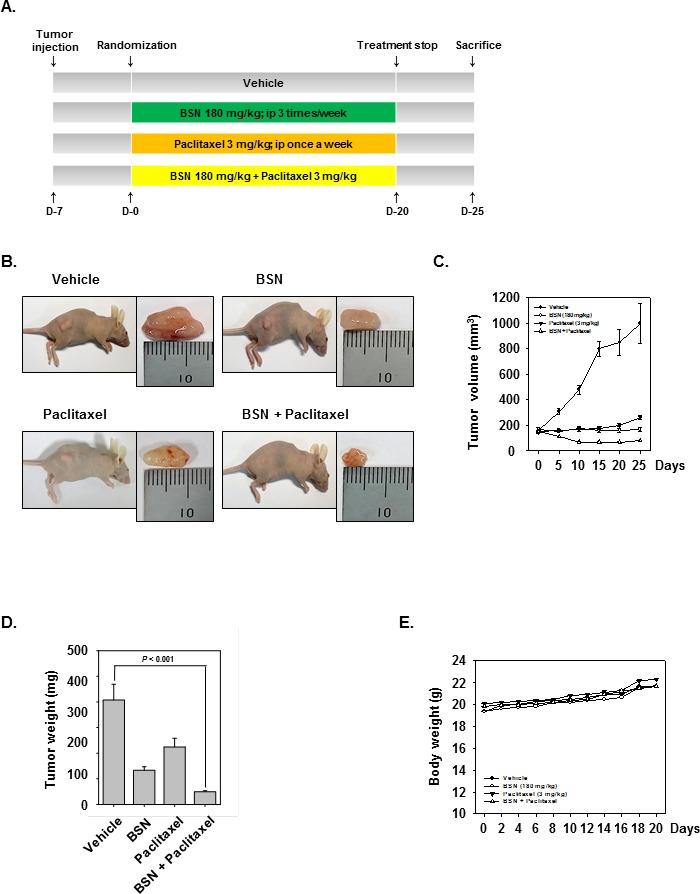
BSN potentiates the antitumor effects of paclitaxel in NSCLC xenograft mouse model (A) A549 cells (1× 10^7^ cells/mice) were injected subcutaneously into the right flank of the mice. The animals were randomized after 1 week of tumor cell injection into four groups based on tumor volume. Group I was treated with PBS (100 μl; i.p.; 3 times/week), group II was treated with BSN alone (180 mg/kg; i.p.; 3 times/week), group III was treated with paclitaxel alone (3 mg/kg; i.p.; once a week), and group IV with a combination of BSN (180 mg/kg; i.p.; 3 times/week) and paclitaxel (3 mg/kg; i.p.; once a week) (n = 8). (B) Necropsy photographs of mice bearing subcutaneously implanted NSCLC tumor. (C) The tumor diameters were measured at 5-day intervals with Digimatic caliper, and the tumor volumes were calculated using the formula V = 4/3 πr^3^ (n = 8). (D) Tumor volumes (mean ± SE) calculated using the formula V = 4/3 πr^3^ (n = 8) after tumor diameters were measured on the last day of the experiment at autopsy using Digimatic caliper. (E). Body weight changes in BSN and paclitaxel treated mice. There was no significant difference in body weight between the control and the treatment groups.

We examined the therapeutic potential of BSN and paclitaxel either alone or in combination on the growth of subcutaneously implanted human lung cancer cells in nude mice. A week after implantation, the animals were randomized into 4 treatment groups based on tumor volume. Treatment was started 1 week after tumor cell implantation and was continued up to 20 days, in accordance with the experimental protocol (Fig. 6A). The tumor diameters were measured at 5-day intervals. Animals were killed 32 days after tumor cell injection and 25 days after the treatment start date, and the tumors were excised and the tumor diameters were measured. We found that BSN alone when given at 180 mg/kg very effective inhibited the growth of the tumor when compared with control. Paclitaxel alone was also significantly when compared with control. The combination of the two agents was more effective in reducing the tumor burden (Fig. 6B and C). The tumor weight (Fig. 6D) in the combination of BSN and paclitaxel group was significantly lower than BSN alone group or paclitaxel alone group. Furthermore, the BSN and paclitaxel did not affect the body weight of mice (Fig. 6E).

### BSN suppresses the growth of human NSCLC *in vivo* and inhibits STAT3 activation from tumor tissues

**FIGURE 7:**
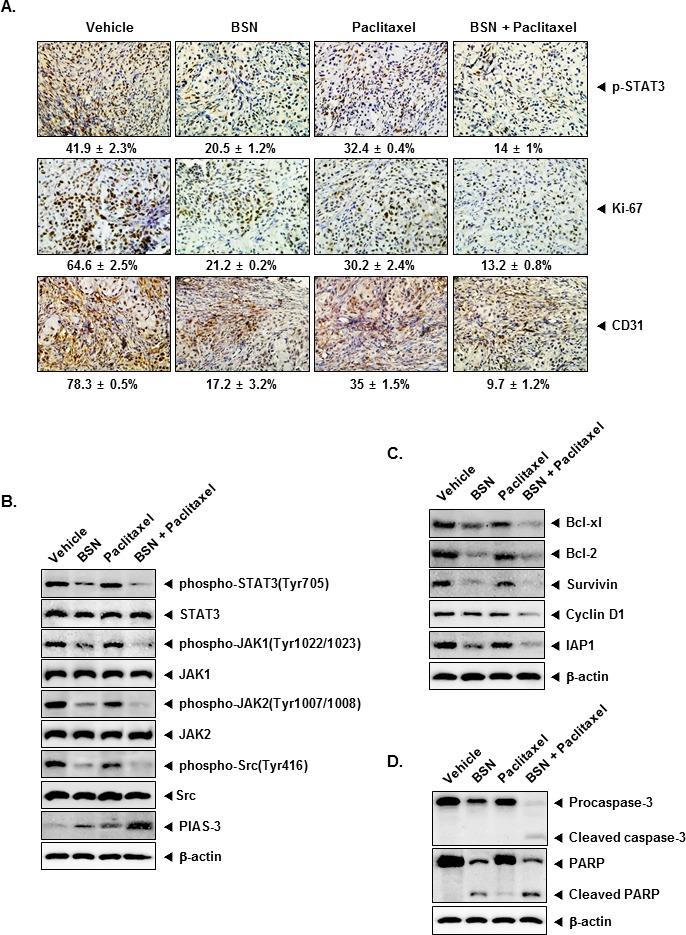
BSN enhances the effect of paclitaxel against the expression of markers of proliferation and angiogenesis in lung cancer tissues (A) Immunohistochemical analysis of phospho-STAT3 showed the inhibition inexpression of phospho-STAT3 in BSN either alone or in combination with paclitaxel-treated samples, as compared with control group (upper panels). Percentage indicates positive staining for the given biomarker. The photographs were taken at the magnification of 40. Immunohistochemical analysis of proliferation marker Ki-67 indicates the inhibition of lung cancer cell proliferation in BSN either alone or in combination with paclitaxel-treated groups of animals (middle panels). Immunohistochemical analysis of CD31 for microvessel density in lung cancer tumors indicates the inhibition of angiogenesis by either BSN alone and in combination with paclitaxel (lower panels). (B) Western blot analysis showed the inhibition of phospho-STAT3 (Tyr705), phospho-JAK1 (Tyr1022/1023), phospho-JAK2 (Tyr1007/1008), phospho-Src (Tyr416), and PIAS-3 by BSN either alone or in combination with paclitaxel-treated groups in whole cell extracts from mice tissue. Western samples from three mice in each group were analyzed and representative data are shown. (C) Equal amounts of lysates were analyzed by Western blot analysis using antibodies against cell survival (Bcl-xl, Bcl-2, and Survivin), proliferation (Cyclin D1), anti-apoptotic (IAP1). Western samples from three mice in each group were analyzed and representative data are shown. (D) Equal amounts of lysates were analyzed by Western blot analysis using antibodies against Procaspase-3, cleaved caspase-3, PARP, and cleaved PARP. The same blots were stripped and reprobed with β-actin antibody to verify equal protein loading. Western samples from three mice in each group were analyzed and representative data are shown.

We also tested the antitumor potential of BSN and paclitaxel either alone or in combination *in vivo* via intraperitoneal administration in a subcutaneous model of human NSCLC using A549 cells. We evaluated the effect of BSN and paclitaxel on constitutive phospho-STAT3 level in NSCLC tumor tissues by immunohistochemical analysis and found that BSN and paclitaxel alone significantly downregulated the expression of phospho-STAT3 in tumor tissues compared with the control group, and the combination of these two was significantly more effective (Fig. 7A, upper panels). The Ki-67-positive index is used as a marker for cell proliferation, and the CD31 index is a biomarker for microvessel density. We found that BSN and paclitaxel downregulated the expression of these biomarkers. BSN and paclitaxel alone significantly inhibited the expression of Ki-67 in tumor tissues compared with the control group, and the combination of these two was significantly more effective (Fig. 7A, middle panels). The results also showed that combination of BSN and paclitaxel remarkably suppressed the expression of CD31 when compared with the control (Fig. 7A, lower panels).

### BSN suppressed the activation of transcription factor STAT3 in NSCLC tumor tissues

We investigated whether BSN either alone or in combination with paclitaxel can affect STAT3 activation in NSCLC tumor tissues. We found that BSN either alone or in combination with paclitaxel moderately suppressed the activated STAT3 by inhibiting phosphorylation at Tyr705. The results showed that treatment with combination of BSN and paclitaxel substantially inhibited constitutive STAT3 activation. We next examined whether BSN either alone or in combination with paclitaxel inhibits p-JAK1, p-JAK2, and p-Src in NSCLC tumor tissues. Because p-JAK1, p-JAK2, and p-Src has also been linked with STAT3 activation. The results showed that treatment with combination of BSN and paclitaxel strongly inhibited constitutive p-JAK1, p-JAK2, and p-Src in NSCLC tumor tissues. Furthermore, combination with BSN and paclitaxel led to an increased expression of PIAS-3 in the tumor tissues (Fig. 7B).

### BSN inhibited the STAT3-regulated gene products in NSCLC tumor tissues

STAT3 activation has been shown to regulate the expression of various gene products involved in cell cycle regulator protein (Cyclin D1), anti-apoptotic proteins (Bcl-xl, Bcl-2, IAP1, and Survivin). Whether BSN and paclitaxel can modulate the expression of these STAT3-regulated gene products in tumor tissues was also examined by Western blot analysis. We found that treatment with combination of BSN and paclitaxel was effective in down-regulating the overexpression of various gene products regulated by STAT3 (Fig. 7C). In addition, caspase-3 activation and PARP cleavage were further increased by co-treatment of BSN and paclitaxel in NSCLC tumor tissues (Fig. 7D).

## DISCUSSION

The aim of this study was to determine whether BSN exerts its anti-cancer effects through modulation of the negative regulators of STAT3 signaling pathway in human lung carcinoma cells. We found that this agent suppressed both constitutive and IL-6-inducible STAT3 activation in the cells in parallel with the inhibition of JAK1, JAK2, and c-Src activation. Surprisingly, BSN triggered the expression of PIAS-3 protein, whereas expression of SOCS-3 was suppressed. Moreover, the knockdown of PIAS-3 using siRNA suppressed the induction of PIAS-3 and reversed the inhibition of STAT3 activation, and resulted in increased cell viability and decreased apoptotic effect. Overexpression of SOCS-3 in BSN-treated cells increased expression of STAT3 phosphorylation, cell viability, and anti-apoptotic effect. BSN down-regulated the expression of various STAT3-regulated gene products, including Bcl-xl, Bcl-2, Survivin, Cyclin D1, IAP1/2, VEGF, MMP-9, and COX-2. It also caused the inhibition of proliferation, increased accumulation of cells in sub-G1 phase, and significantly enhanced the apoptotic effects of paclitaxel in A549 cells. Intraperitoneal injection of BSN and paclitaxel into athymic *nu/nu* female mice bearing subcutaneous A549 xenografts resulted in significant suppression of tumor progression and inhibition of STAT3 activation in tumor tissues.

We found for the first time that BSN could suppress both constitutive and IL-6-induced STAT3 phosphorylation at both Tyr705 and Ser727 in A549 cells and that these effects were cell-type specific, as BSN had no effect on STAT3 phosphorylation in U266, DU145, K562, and SCC4 cell lines. We also observed that BSN inhibited nuclear translocation and DNA binding activity of STAT3. How BSN affects STAT3 activation was also examined in detail. The effects of BSN on STAT3 phosphorylation correlated with the suppression of upstream protein tyrosine kinases JAK1, JAK2, and c-Src in A549 cells. STAT3 phosphorylation has been closely associated with transformation and proliferation of tumor cells [3, 30]. All Src-transformed cell lines have persistently led to the STAT3 activation, and dominant-negative STAT3 abrogates transformation [31, 32]. Recent reports showed constitutive STAT3 activation in lung cancer cell lines and tissues [33-35]. Antisense STAT3 oligonucleotides have been found to induce complete loss of STAT3 DNA-binding activity and apoptosis in A549 cells [36].

We found evidence that the BSN-induced inhibition of STAT3 activation involves two negative regulator of STAT3 namely PIAS-3 and SOCS-3. PIAS3 has been known to control STAT3 transcriptional activity in lung cancer cells through affecting its DNA transcriptional properties and STAT3 phosphorylation [37, 38]. It has also been reported that the overexpression of PIAS-3 inhibits STAT3 transcriptional activity and consistently decreased proliferation of NSCLC cell lines [38]. Absence of PIAS-3 has been shown to enhance STAT3 transcriptional activity and subsequent cell proliferation in glioblastoma multiforme tumors [39]. Loss of PIAS-3 following transfection with PIAS-3 siRNA abolished the STAT3 inhibitory effects of BSN and we further found that deletion of PIAS-3 reversed BSN-induced cell growth inhibition and induction of apoptosis. Additionally, our results showed that the high expression of SOCS-3 was noted at the basal level in A549 cells. These results are in agreement with results showing that the SOCS-3 expression was highly elevated in human breast cancer [40], melanoma tissues [41], and primary lymphoma cells [42]. The *marked* reduction in the *expression* of *SOCS-3* following BSN treatment of A549 cells resulted in the marked suppression of STAT3 activation, and overexpression of SOCS-3 by using SOCS-3 plasmid abrogated BSN-mediated STAT3 inhibition, cell growth inhibition, and induction of apoptosis.

Our previous study has demonstrated that BSN suppressed the constitutive activation of PI3K/Akt/mTOR/S6K1 pathway, which correlated with the induction of apoptosis in PC-3 cells [29]. Here, we found that BSN inhibited the phosphorylation of STAT3 at both Tyr705 and Ser727 in A549 cells. It has been known that STAT3 phosphorylation at serine residue is mediated by the rapamycin target mTOR in ciliary neurotrophic factor signaling [43]. JAK/STAT pathway is one of the important downstream routes for epidermal growth factor receptor (EGFR) signaling [44], whereas PI3K is one of the downstream signaling molecules of the EGFR and plays a role in the proliferation or invasion of tumor cells [45, 46], thereby further implicating important correlation between STAT3 and PI3K/Akt signaling cascades. Moreover, BSN also suppressed the constitutive ERK activation that is known to be required for STAT3 phosphorylation on serine 727 [47].

We also demonstrate that BSN inhibited the expression of various STAT3-regulated gene products, such as anti-apoptotic (Bcl-xl, Bcl-2, Survivin, and IAP1/2), proliferative (Cyclin D1), metastatic (MMP-9 and COX-2), and angiogenetic (VEGF). Constitutive STAT3 activation has been known to *induce resistance to apoptosis* [48], possibly through up-regulation of Bcl-2, Bcl-xl, IAP1/2, and Survivin expression [49]. The down-regulation of Cyclin D1 expression by BSN associated with the suppression in proliferation and accumulation of cells in the sub-G1 phase of the cell cycle, suggesting that Cyclin D1 plays an important role in the observed anti-proliferative effect of BSN. We also found that BSN significantly inhibited tumor cell invasion activity in A549 cells, which may be explained by its ability to negatively regulate the expression of MMP-9 and COX-2 proteins. Bcl-2 and Bcl-xl expression are mainly regulated by STAT3 pathway, and these proteins are overexpressed in lung cancer cells [50, 51]. The downregulation of Bcl-2, Bcl-xl and survivin proteins could account for BSN's ability to induce substantial apoptosis in A549 cells.

Although paclitaxel is widely used for the treatment of NSCLC, this drug has severe side effects and patients frequently develop chemoresistance [52, 53]. Besides, paclitaxel has shown synergistic interaction with various classes of targeted therapeutic agents and is at present being evaluated in the neo-adjuvant and adjuvant treatment settings for early stage NSCLC [53]. We also noted that BSN can synergistically improve paclitaxel sensitivity in A549 cells. Interestingly, BSN was found to exert synergistic cytotoxic effect at a low concentration of 25 μM and higher concentrations like 50 or 75 μM did not significantly affect the paclitaxel sensitivity. The combinational treatment potentiated paclitaxel-induced apoptosis through the downregulation of various STAT3-regulated gene products. We further observed that BSN and paclitaxel co-treatment significantly suppressed tumor growth in a xenograft lung cancer model and also substantially downregulated the expression of various STAT3 regulated genes in mice tissues. In addition, our preclinical studies imply that the potential of combining BSN with paclitaxel to reduce the harmful side effect of chemotherapy during the treatment of lung cancer.

Overall, our results indicate for the first time that BSN can inhibit constitutive and inducible STAT3 signaling pathway through modulation of PIAS-3 and SOCS-3 proteins, as well as can enhance the effects of paclitaxel through the downregulation of gene products that mediate tumor cell survival, proliferation, invasion, and metastasis in human lung cancer.

## MATERIALS AND METHODS

### Reagents

Brassinin (BSN, Fig. 1A) was purchased from LKT laboratories (Minneapolis, MN). Stock solution of BSN (100 mM) was prepared in dimethyl sulfoxide, stored at −80°C, and diluted in cell culture medium for use. RPMI 1640, fetal bovine serum (FBS), and antibiotic-antimycotic mixture were obtained from Thermo Fisher Scientific Inc. (Waltham, MA). Trypan blue was obtained from GIBCO (Grand Island, NY). 3-(4,5-Dimethylthiazol-2-yl)-2,5-diphenyltetrazolium bromide (MTT) was purchased from Sigma-Aldrich (St. Louis, MO). SDS, Tris, Glycine, and NaCl were obtained from Sigma-Aldrich (St. Louis, MO). Bovine serum albumin was purchased from Biosesang (Sungnam, Korea). Rabbit polyclonal antibody against STAT3 and mouse monoclonal antibodies against phospho-STAT3 (Tyr705), phospho-STAT3 (Ser727), Bcl-2, Bcl-xl, PIAS-3, SOCS-3, Caspase-3, Cyclin D1, IAP1, IAP2, COX-2, MMP-9, Survivin, VEGF, PARP, phospho-Akt (Ser473), Akt, Ki-67, CD31, β-actin, goat anti-rabbit IgG-HRP, and goat anti-mouse IgG-HRP were purchased from Santa Cruz Biotechnology (Santa Cruz, CA). Antibodies against phospho-Src (Ty416), Src, phospho-JAK1 (Tyr1022/1023), JAK1, phospho-JAK2 (Tyr1007/1008), JAK2, phospho-ERK (Thr202/Tyr204), and ERK were purchased from Cell signaling Technology (Beverly, MA). Paclitaxel was obtained from Sigma-Aldrich (St. Louis, MO). pCMV, pCMV-SOCS-3, pMXS-gw, and pMXs-STAT3C were obtained from Addgene (Cambridge, MA). Whole-cell lysates of tumor tissues were obtained with T-PER Tissue Protein Extraction Reagent (Pierce, Rockford, USA).

### Cell lines and culture conditions

Human multiple myeloma cell lines U266, human prostate carcinoma DU145, human lung cancer cell lines A549, H1299, and H460, human chronic myelogenous leukemia cell line K562, human squamous cell carcinoma SCC4, and mouse embryonic fibroblast (MEF) were obtained from the American Type Culture Collection (Manassas, VA, USA). Human lung cancer cell line PC-9 was purchased from Immuno-Biological Laboratories (Gunma, Japan). The U266, DU145, A549, H1299, H460, PC-9, and K562 cells were cultured in RPMI 1640 supplemented with 10% fetal bovine serum and 1% penicillin-streptomycin. MEF cells were cultured in DMEM supplemented with 10% fetal bovine serum and 1% penicillin-streptomycin. SCC4 cells were cultured in DMEM supplemented with 10% fetal bovine serum, 1% penicillin-streptomycin, 1% vitamin solution, and 1% non-essential amino acid solution. All cells maintained at 37°C in a 5% CO_2_ atmosphere. At ~70–90% confluence, the cells were subcultured using 0.05% trypsin/EDTA (Gibco-BRL).

### Western blot analysis

For detection of STAT proteins, BSN-treated whole-cell extracts were lysed in a lysis buffer (20 mM Tris (pH 7.4), 250 mM NaCl, 2 mM EDTA (pH 8.0), 0.1% Triton X-100, 0.01 mg/ml aprotinin, 0.005 mg/ml leupeptin, 0.4 mM phenyl methane sulfonyl fluoride (PMSF), and 4 mM NaVO_4_). The lysates were then spun at 14,000 rpm for 10 min to remove insoluble material and resolved on a 8% SDS-PAGE. After electrophoresis, the proteins were electrotransferred to a nitrocellulose membranes (Pall Corporation, MI, USA), blocked with 5% nonfat milk, and probed with anti-STAT antibodies (1:1000) overnight at 4°C. The blots were washed, exposed to HRP-conjugated secondary antibodies for 2 h, and finally examined by chemiluminescent substrate (GE Healthcare, Waukesha, USA). For the apoptotic study, the appropriate amounts of cell lysates (20 μg) were separated at 8 to 12% SDS-polyacrylamide gel electrophoresis and electro-transferred onto nitrocellulose membranes. The membranes were blocked with 5% nonfat milk in TBST (Tris-buffered saline with 0.1% Tween 20) for 2 h, washed and then incubated with the following primary antibodies: anti-PARP, anti-Caspase-3, anti-Cyclin D1, anti-Survivin, anti-IAP1/2, anti-VEGF, anti-MMP-9, anti-COX-2, anti-Bcl-2, and anti-Bcl-xl (diluted 1:1000 in 5% skim milk in TBST; Santa Cruz Biotechnology, Santa Cruz, CA). The equal loading of samples were controlled using β-actin. Antibodies were incubated overnight at 4°C on a rocking platform. The membranes were then incubated for 2 h with HRP-conjugated anti-mouse IgG or anti-rabbit IgG Abs (diluted 1/5000 in TBST) and the immunoreactive bands were developed using a chemiluminescent substrate.

### EMSA for STAT3-DNA binding

STAT3-DNA binding was analyzed by electrophoretic mobility shift assay (EMSA) using a ^32^P-labeled high-affinity sis-inducible element (hSIE) probe (5′-CTTCATTTCCCGTAAATCCCTAAAGCT-3′ and 5′-AGCTTTAGGGATTTACGGGAAATGA-3′) as previously described [54]. Briefly, nuclear extracts were prepared from BSN-treated cells and incubated with the labeled hSIE probe. The DNA–protein complex formed was separated from free oligonucleotide on 5% native polyacrylamide gels. The dried gels were visualized with a Universial hood II (Bio-rad, Hercules, CA).

### Immunocytochemistry for STAT3 localization

A549 cells were with BSN for 4 h, the cells were fixed in 4% paraformaldehyde (PFA) for 20 min at room temperature and then washed three times in PBS. The cells were permeabilized with 0.2% Triton X-100 in PBS for 20 min, washed three times in PBS, and then blocked with 5% BSA in PBS for 1 h at room temperature. The cells were then incubated overnight at 4°C with anti-STAT3 (1:100; Santa Cruz Biotechnology, Santa Cruz, CA), washed three times, and incubated with Alexa 488-labeled goat anti-rabbit IgG (1:200; Molecular Probes, Eugene, OR) for 1 h at room temperature. Next, the cells were stained with a 1 μg/ml DAPI solution and mounted on glass slides using Fluorescent Mounting Medium (GBI Laboratories, Manchester, UK). Using an Olympus FluoViewFV1000 confocal microscope (Olympus, Tokyo, Japan), DAPI and FITC fluorescence were excited (Ex: 405 nm and 488 nm) and detected (Em: 461 nm and 519 nm) with 2.1% laser transmissivity and 5.0% laser transmissivity, respectively.

### Reverse transcription polymerase chain reaction (RT-PCR)

Cells were washed and suspended in Trizol reagent. Total RNA was extracted according to the manufacturer's instructions (Invitrogen, Life Technologies). One microgram of total RNA was converted to cDNA by superscript reverse transcriptase and then amplified by Platinum Taq polymerase using superscript one step reverse transcription-PCR (RT-PCR) kit (Invitrogen, Carlsbad, CA). The relative expressions of PIAS-3 and SOCS-3 were analyzed using MyGene^TM^ Series Peltier Thermal Cycler (Model MG96G, LongGene, Hangzhou, PRC) with glyceraldehyde-3-phosphate dehydrogenase (GAPDH) as an internal control. The reaction was performed at 50°C for 30 min, 94°C for 2 min, and 30 cycles of 94°C for 15 s, 60°C for 30 s, and 72°C for 1 min, with extension at 72°C for 10 min. PCR products were run on 1% agarose gel and then stained with loading star (Dynebio, Gyeonggi, Korea). Stained bands were visualized under UV light and photographed.

### Transfection of plasmids

We investigated the ability of commercially available electroporation systems, the Neon™ Transfection System (Invitrogen, Carlsbad, CA). Transfection efficiency was measured by Western blot analysis. A549 and MEF cells were prepared for transfection after cells were resuspended with 120 μl of Neon Resuspension Buffer R for every one million cells. For each electroporation, A549 cells with 50 nM of PIAS-3 siRNA, scrambled siRNA, 1 μg of pCMV-SOCS-3 or pCMV plasmids, MEF cells with 1 μg of pMXs-STAT3C or pMXs-gw plasmids were aliquoted into a sterile microcentrifuge tube. A Neon Tip was inserted into the Neon Pipette and the mixture was aspirated into the tip avoiding air bubbles. The Neon Pipette was then inserted into the Neon Tube containing 3 ml of Neon Electrolytic Buffer E in the Neon Pipette Station. A549 cells were pulsed twice with a voltage of 1,200 and a width of 30. MEF cells were pulsed once with a voltage of 1,350 and a width of 30. After 48 h of transfection, A549 and MEF cells were treated with 300 μM of BSN for 4 h or 24 h. Then whole-cell extracts were prepared for PIAS-3, SOCS-3, phospho-STAT3 (Tyr705), STAT3, PARP, and β-actin analysis by Western blotting.

### MTT assay

A549 cells were seeded at a density of 1×10^4^ cells/well in 96-well plates. The cells were incubated with 300 μM of BSN. After 24 h incubation, 20 μl of MTT (2 mg/ml) was added to each well. After incubation at 37°C for 2 h, extraction buffer (20% SDS and 50% dimethylformamide) was added to the cells. The cells were incubated overnight at 37°C, and the absorbance was then measured at 570 nm by a microplate reader (Bio-Rad, Hercules, CA).

### Live and dead assay

To measure apoptosis, we used the Live and Dead assay (Invitrogen, Carlsbad, CA), which determines intracellular esterase activity and plasma membrane integrity. This assay employs calcein, a polyanionic dye, which is retained within the live cells and provides green fluorescence. It also employs the ethidium monomer dye (red fluorescence), which can enter the cells only through damaged membranes and bind to nucleic acids but is excluded by the intact plasma membrane of live cells. Briefly, A549 cells were seeded at a density of 3×10^4^ cells/well in 8-well slide chamber. The cells were incubated with 300 μM of BSN for 24 h. Cells are stained with the Live and Dead reagent (5 μM ethidium homodimer, 5 μM calcein-AM) and then incubated at 37°C for 30 min. Cells were analyzed under an Olympus FluoViewFV1000 confocal microscope (Olympus, Tokyo, Japan).

### Monitoring of cell growth with the RTCA MP Instrument

Cell growth behavior was continuously monitored for 72 h using the xCELLigence RTCA MP Instrument (Roche Diagnostics GmbH, Germany). Background impedance was measured in 100 μl cell culture medium per well. The final volume was adjusted to 200 μl cell culture medium, including 5 × 10^3^ cells/well. After plating, impedance was recorded in 15 min intervals. All experiments were performed in triplicates. Cell Index (CI) values were normalized to the time point of 100 and 300 μM of BSN administration (referred to as normalized CI).

### Invasion assay

We employed the Roche xCELLigence Real-Time Cell Analyzer (RTCA) DP instrument (Roche Diagnostics GmbH, Germany) to measure cellular invasion. The RTCA DP instrument uses the CIM (cellular invasion/migration)-Plate 16, which features microelectronic sensors integrated onto the underside of the microporous polyethylene terephthalate (PET) membrane of a Boyden-like chamber. For invasion experiments, the top chamber of the CIM-Plate 16 was coated with matrigel (BD Biosciences, Becton-Dickinson, Franklin Lakes, NJ) before addition of the medium to the bottom chamber. The CIM-Plate 16 was assembled by placing the top chamber onto the bottom chamber and snapping the two together. Serum-free medium was placed in the top chamber to hydrate and pre-incubate the membrane for 1 h in the CO_2_ incubator at 37°C before obtaining a background measurement. Cells were lightly trypsinized, pelleted and resuspended at the indicated cell densities in serum-free medium. Once the CIM-Plate 16 has been equilibrated, it was placed in the RTCA DP station and the background cell index values were measured. The CIM-Plate 16 was then removed from the RTCA DP station and the cells are added to the top chamber at the desired density.

### Cell cycle analysis

To determine apoptosis, cell cycle analysis was performed using propidium iodide. A549 cells were seeded onto 6-wellplates at a density of 1 × 10^6^ cells/well and incubated for one day. After treatment with 300 μM of BSN for 24 h, the cells were collected and washed with 1× PBS. Cell pellets were fixed in 70% cold ethanol overnight at −20°C. The fixed cells were resuspended in 1× PBS containing 1 mg/ml RNase A, incubated for 1 h at 37°C incubation. Cells were then washed, resuspended, and stained in PBS containing 25 μg/ml of propidium iodide for 30 min at room temperature in the dark. The DNA contents of the stained cells were analyzed using Cell Quest Software with a FACScan Calibur flowcytometry (BD Biosciences, Becton-Dickinson, Franklin Lakes, NJ).

### Annexin V assay

One of the early indicators of apoptosis is the rapid translocation and accumulation of the membrane phospholipid phosphatidylserine from the cell's cytoplasmic interface to the extracellular surface. This loss of membrane asymmetry can be detected using the binding properties of annexin V. A549 cells were plated at a density of 1 × 10^6^ cells/well in 6-well plate. The cells were treated with 300 μM of BSN for 24 h. After treatment, the cells were collected and washed with cold PBS. Treated sample were assayed for phosphatidylserine exposure by using an annexin V-FITC Apoptosis Detection Kit (Bio-Rad, Hercules, CA) according to the manufacturer's instructions. Stained samples were analyzed by a flow cytometer (FACScanCalibur, BD Biosciences, Becton-Dickinson, Franklin Lakes, NJ). Acquisition and analysis of the data were performed using Cell Quest 3.0f software.

### Combination therapy studies

A549 cells were seeded and cotreated with BSN (0, 25, 50, or 75 μM) and paclitaxel (0, 1, 2.5, or 5 nM) for an additional 24 h. After treatment, cells were collected and washed with chilled PBS, and the treated samples were examined using a MTT assay and Western blotting.

### Animal study

All procedures involving animals were reviewed and approved by Kyung Hee University Institutional Animal Care and Use committee [KHUASP(SE)-13-044]. Six-week-old athymic *nu/nu* female mice were purchased from Orientbio Inc. (Sungnam, Korea). The animals were housed (8 mice/cage) in the standard mice plexiglass cages in a room maintained at constant temperature and humidity under 12 h light and dark cycle and fed with regular autoclaved mouse chow with water *ad libitum*. None of the mice exhibited any lesions and all were tested pathogen-free. Before initiating the experiment, we acclimatized all mice to a pulverized diet for 3 days.

### Subcutaneous implantation of A549 cells

A549 cells were injected subcutaneously into the mice as described previously [55]. In brief, A549 cells were harvested from subconfluent cultures, washed once in serum-free medium, and resuspended in PBS. Only suspensions consisting of single cells, with > 90% viability, were used for the injections. A549 cells [1 × 10^7^ cells/100 μl PBS:Matrigel (1:1)] were injected subcutaneously into the right flank. To prevent leakage, a cotton swab was held cautiously for 1 minute over the site of injection.

### Experimental protocol

After 1 week of implantation, tumor diameters were measured using Digimatic caliper (Mitutoyo Company, Japan). When tumors have reached 0.25 cm in diameter, the mice were randomized into the following treatment groups (n = 8/group) based on the tumor volume. Group I (control) was treated with corn oil (100 μl; i.p.;3 times/week), group II was treated with BSN alone (180 mg/kg; i.p.; 3 times/week), group III was treated with paclitaxel alone (3 mg/kg; i.p.; once a week), and group IV with a combination of BSN (180 mg/kg; i.p.; 3 times/week) and paclitaxel (3 mg/kg; i.p.; once a week) (n = 8). Treatment was continued for up to 20 days from the date of randomization (Day 0). The tumor volume was measured at 5-day intervals. The mice were killed 25 days after randomization. The tumors were carefully excised and measured to calculate tumor volume. The tumor volume was derived using the formula V = 4/3 πr^3^, where r is the mean of the 3 dimensions (length, width, and depth). Half of the tumor tissue was fixed in formalin and embedded in paraffin for immunohistochemistry and routine hematoxylin and eosin (H&E) staining. The other half was snap frozen in liquid nitrogen and stored at −80°C.

### Western blot analysis for tumor tissues

NSCLC tumor tissues (75–100 mg) from control and experimental mice were minced and incubated on ice for 30 minutes in 0.5 ml of T-PER Tissue Protein Extraction Reagent. The minced tissue was centrifuged at 16,000 × g at 4°C for 20 minutes. The proteins were then fractionated by SDS-PAGE, electrotransferred to nitrocellulose membranes, blotted with each antibody, and detected by enhanced chemiluminescent substrate (GE Healthcare, Waukesha, USA).

### Immunohistochemical analysis of NSCLC tumor samples

Solid tumors from control and various treatment groups were fixed with 10% neutral buffered formalin (BBC Biochemical, USA), processed and embedded in paraffin. Sections were cut and deparaffinized in xylene, and dehydrated in graded alcohol and finally hydrated in water. Antigen retrieval was performed by boiling the slide in 10 mM sodium citrate (pH 6.0) for 30 minutes. Immunohistochemistry was performed following manufacturer instructions (Vector Laboratories ImmPRESSTM REAGENT KIT). Briefly, endogenous peroxidases were quenched with 3% hydrogen peroxide. Non-specific binding was blocked by incubation in the blocking reagent in the ImmPRESSTM REAGENT KIT (Vector Laboratories, Burlingame, CA) according to the manufacturer's instructions. Sections were incubated overnight with primary antibody: phospho-STAT3, anti-Ki-67 and CD31 (at 1:100 dilutions). Slides were subsequently washed several times in phosphate-buffered saline (PBS) and were incubated with ImmPRESSTM reagent according to the manufacturer's instructions. Immunoreactive species were detected using 3, 3-diaminobenzidine tetrahydrochloride (DAB) as a substrate. Sections were counterstained with Gill's hematoxylin and mounted under glass cover slips. Images were taken using an Olympus BX51 microscope (magnification, 40×). Positive cells (brown) were quantitated using the Image-Pro plus 6.0 software package (Media Cybernetics, Inc.).

### Statistical analysis

The results were expressed as means ± SD, and an analysis of variance (ANOVA) with Bonferroni's test was used for the statistical analysis of multiple comparisons of data. *P-*value of 0.05 or less was considered as significant. To obtain the evidence of a synergistic effect between BSN and Paclitaxel, the combination index (CI) was determined by the Chou–Talalay method and calcusyn software (Biosoft, Ferguson, MO).
